# Polymorphisms for Defence and Virulence in the *Arabidopsis thaliana*–*Cucumber mosaic virus* Interaction Are Expressed in the Host’s Native Habitat

**DOI:** 10.3390/v18050494

**Published:** 2026-04-23

**Authors:** Israel Pagán, Rafael de Andrés-Torán, Nuria Montes, Aurora Fraile, Fernando García-Arenal

**Affiliations:** Centro de Biotecnología y Genómica de Plantas UPM-INIA/CSIC, Departamento de Biotecnología-Biología Vegetal, E.T.S. Ingeniería Agronómica, Alimentaria y de Biosistemas, Universidad Politécnica de Madrid, Campus de Montegancedo, UPM, 28223 Pozuelo de Alarcón, Madrid, Spain; jesusisrael.pagan@upm.es (I.P.); rafaelde.andres@upm.es (R.d.A.-T.); nuria.montes.casado@gmail.com (N.M.); aurora.fraile@upm.es (A.F.)

**Keywords:** *Arabidopsis thaliana*, *Cucumber mosaic virus*, field conditions, native habitat, resistance, tolerance, virulence, plant–virus interaction

## Abstract

Plant defences are assumed to evolve in response to the negative effects of virus infection on plant fitness (virulence), and to drive plant–virus coevolution. However, viruses are not always antagonistic symbionts of plants, and the expression of defence traits is environment-dependent. Thus, understanding plant–virus interactions requires analysing the expression of defence traits in the host’s native habitat. Here we analyse the effect of cucumber mosaic virus (CMV) infection, and the expression of resistance and tolerance in the native habitat of a wild *Arabidopsis thaliana* population. Plants from ten genotypes from that population, which have been shown to differ in resistance and tolerance to CMV in a greenhouse, were inoculated with an Arabidopsis isolate of CMV and transplanted to their habitat. Resistance was rated based on virus accumulation in leaves, and tolerance was rated based on the effect of infection on plant fecundity relative to virus accumulation. Consistent with the greenhouse assays, virulence depended on the host genotype, and polymorphisms for resistance and tolerance were expressed in the field, supporting the validity of the conclusions from the greenhouse assays. Our results also support theoretical predictions on the relationships between pathogen multiplication and virulence and between resistance and tolerance.

## 1. Introduction

Knowledge of the conditions in which plant defences against viruses are expressed is central to understanding plant–virus interactions, their evolution, and the use of such defences to control viral diseases of crops. As for other pathogens, the two major defences of plants against viruses are resistance, i.e., the host’s ability to limit pathogen infection and multiplication, and tolerance, i.e., the host’s ability to reduce the negative effects of infection at a given pathogen load [1,2,3]. Resistance and tolerance represent two fundamentally different strategies of plant defence. In addition, because host resources that can be allocated to defence are limited, resistance and tolerance are assumed to be negatively correlated across host genotypes and to evolve under different scenarios [4,5,6,7,8]. Hence, they are predicted to lead to different outcomes of plant–pathogen interactions [2,9,10,11]. Both resistance and tolerance are assumed to have evolved as a plant’s response to the negative effect of pathogen infection on their fitness, i.e., virulence, and to be drivers of host–pathogen coevolution [12,13]. Despite the rich theory on the evolution of host defences and host–pathogen coevolution, evidence of the negative effects of pathogen infection on plant fitness and of plant–pathogen coevolution in non-agricultural pathosystems, where both partners may evolve with no human intervention, is scant and comes mostly from the study of plant interactions with fungi, oomycetes or bacteria [14,15]. This lack of evidence is particularly noticeable for plant viruses, as it is unclear if or when virus infection imposes a selective force on plant populations [16,17]. Addressing this question requires analysing the expression of defence traits under field conditions, which may differ from the expression under controlled experiments [18].

The negative effects of virus infection on the fitness of wild plants under field conditions have been shown for a variety of systems [19,20,21,22,23,24,25,26,27,28], and polymorphisms for defence traits in wild plant populations have been demonstrated in assays under controlled conditions [29,30,31,32,33,34]. However, disease is not the universal outcome of plant–virus interactions [35,36], and a large fraction of infections in wild plant communities are asymptomatic [36,37,38,39,40,41,42,43,44]. Experiments under controlled conditions have shown that virus infection can be beneficial to plants under a wide range of abiotic stresses [45,46,47], which may favour the evolution of viruses to mutualism [48]; it is widely accepted that viruses can be conditional mutualists or pleiotropic parasites of plants [49,50]. This could be particularly relevant in wild plant communities, where growth conditions are often less favourable than in human-managed environments such as crop fields. Accordingly, the positive effects of virus infections in plants growing in their natural wild communities have been shown in a few systems [51,52]. Thus, it is not obvious that the expression of antiviral plant defences is the norm in their native habitats.

Here we analyse the effects of a virus infection and whether plant defence traits are expressed under field conditions, using the system *Cucumovirus CMV* (cucumber mosaic virus, *Bromoviridae*) (CMV)—*Arabidopsis thaliana* L. Heynh. (Brassicaceae) (Arabidopsis). Arabidopsis, a model plant for evolutionary ecology [53], is an annual plant with two developmental stages—the vegetative growth period, which produces a rosette of leaves, and the reproductive period, in which the inflorescence grows and flowers are produced and develop into siliques continuously until plant senescence. Arabidopsis is found in a large variety of habitats in the Iberian Peninsula, where it has a large genetic diversity, translated into phenotypic diversity for a wide variety of traits [54,55]. In Iberian populations, plants flower in spring and have two major cohorts depending on whether they germinate in the autumn/winter and overwinter as rosettes or germinate in the spring [44,56,57]. All aspects of the biology of CMV have been reviewed recently [58]. Briefly, CMV is an RNA virus with a large host range of more than 1200 mono- and dicotyledon species that is horizontally transmitted by aphids in a non-persistent manner. CMV is also transmitted vertically through the seed, with rates that in Arabidopsis vary between 1 and 8% depending on the virus genotype–host genotype interaction [59,60,61]. CMV isolates are highly diverse and have been classified into subgroups IA, IB and II, based on the nucleotide sequence similarity of their genomic RNAs. In central Spain, isolates of subgroup IA are the most prevalent [62], and metagenomic analyses revealed the presence of only subgroup IA isolates in the region and at the time of the present study, with a low genetic diversity [63].

Ten years of field surveys have shown a high prevalence of CMV subgroup IA isolates in six wild Arabidopsis populations from central Spain, of up to 80% according to population and year [38], indicating that the Arabidopsis–CMV interaction is significant in nature. Analysis under controlled conditions of the interaction of 21 wild genotypes of Arabidopsis representing the variation of the species in Eurasia with three CMV strains showed that both quantitative resistance and tolerance to CMV depended on the host genotype, and both traits had moderate to high heritability [64,65]. The analysis of the interaction of 185 individuals representing 76 wild Iberian Arabidopsis populations with two CMV isolates from these populations showed substantial genetic variation within and between host populations for both quantitative resistance and tolerance, and strongly suggested that CMV infection selects for defence in Arabidopsis, implying that CMV infection reduces host fitness in the environment of the Arabidopsis populations studied [32]. However, highly effective resistance associated with *R* genes and expressed as hypersensitivity was not found in any of the 76 Iberian populations, which may suggest that selection for defence is not strong in these populations [32]. Indeed, the only in situ analysis of the effects of CMV infection on the life history traits of Arabidopsis plants involving two wild populations showed that the interaction moved along the mutualist–antagonist continuum, and that the effects of infection on plant fitness may be positive, neutral or negative depending on the population, the plant cohort and the time of infection relative to plant development [44]. In this study, CMV infection was due to natural transmission, and the analysed infected and non-infected plants were not genotyped, such that neither partner in the interaction was genetically characterised. Here we report on a field experiment in which individual plants of ten genotypes from a central Spain population of Arabidopsis were infected with a CMV isolate derived from this host, transplanted to the site of the original Arabidopsis population and monitored for the effects of virus infection and the expression of defence traits.

## 2. Materials and Methods

### 2.1. Virus Isolates, Arabidopsis thaliana Genotypes and Plant Inoculation

Four cucumber mosaic virus Subgroup IA isolates were obtained between 2008 and 2012 from individual Arabidopsis plants collected in three wild populations located in a 150 km N-S transect in central Spain at Ciruelos de Coca, Rascafría and Las Rozas (isolates Cdc1-CMV, Cdc2-CMV, Ras-CMV and Lro-CMV). Leaf sap from field-infected plants was inoculated into *Chenopodium quinoa* Wild. and isolates were biologically cloned by two local lesion passages in this host [32]. CMV isolates were multiplied in *Nicotiana clevelandii* (A. Gray), virus particles were purified as in [66] and RNA was obtained from purified particles after SDS disruption and phenol extraction. The nucleotide sequence of the genomic RNAs was determined by Sanger sequencing outsourced at STAB VIDA (Caparica, Portugal) on DNA amplicons obtained with the primer pairs indicated in Table S1. Accession numbers for the determined sequences are: PZ315230, PZ315234 and PZ315238, for Cdc1-CMV; PZ315231, PZ315235 and PZ315239, for Cdc2-CMV; PZ315228, PZ315232 and PZ315236, for Lro-CMV; and PZ315229, PZ315233 and PZ315237, for Ras-CMV.

Lro-CMV, the isolate from the population geographically closest to Marjaliza, was used for mechanical inoculation of Arabidopsis plants at the five-leaf stage (stage 1.05 of [67]) with 15 μL of sap from infected *N. clevelandii* leaves in 0.01 M phosphate buffer pH 7.0, 0.2% sodium diethyldithiocarbamate. A total of 15 μL of buffer was applied to mock-inoculated controls.

Ten Arabidopsis genotypes derived from individual plants collected at the Marjaliza (MAR, Spain) wild population were used: MAR2, MAR6, MAR8, MAR10, MAR12, MAR13, MAR15, MAR23, MAR25 and MAR29 [32]. The field-collected plants were propagated by selfing during two generations by the single-seed descent procedure. This allowed reducing potential residual heterozygosity in wild individuals and removing maternal and grand-mother effects. These individuals were previously genotyped for 250 genome-wide SNPs that were segregated in the plant population [54,68]. Seeds were stratified (darkness, 4°C) for 7 days before germination at 25/20°C day/night, 16 h light in a growth chamber (January 2017). Ten-day-old seedlings were transplanted to 46 alveoli trays and moved to the greenhouse, where they were kept (25/20°C day/night, 16 h light) until inoculation. After inoculation, plants were moved to an open plastic tunnel (February 2017) and kept there for acclimatation to open field conditions. On 6 February 2017, one week after inoculation, plants were transplanted into the field, within the area occupied by the Marjaliza Arabidopsis wild population [38], where they remained until the end of the experiment.

### 2.2. Experimental Design

Sixty plants per Arabidopsis genotype were used. Thirty were inoculated with Lro-CMV and the other 30 were mock-inoculated. Plants were divided into four groups of 15 plants, each with 7–8 mock-inoculated and infected plants of each genotype. Plants of each group were transplanted into four different patches, such that plants of all genotypes were transplanted at each patch. Patches were randomly distributed across a 500 m transect within the area of the MAR Arabidopsis wild population.

Plants were visited three times during the experiment: at the vegetative stage (30 March 2017), at the flowering stage (27 April 2017) and at the late fructification stage (when all siliques formed were at the maturation phase) (11 May 2017). Note that in the field, plant development was not synchronised, such that on April 27th some plants had started to produce siliques and on May 11th some plants had already completed their life cycle. For each plant, the maximum rosette diameter (in cm) was quantified at each visit, and the number of siliques and the length (in cm) of up to five siliques were quantified at the second and third visits, as measuring every silique per plant was unpractical. Plant fecundity was estimated as the total length of siliques per plant (number of siliques × average silique length), which is highly correlated with the number of seeds both under controlled and field growth conditions [44,69]. In the first visit, 4 mm diameter disks of rosette leaves were sampled in all plants for CMV detection and quantification (File S1).

Mortality rates during the experiment ranged from 13% for MAR10 to 43% for MAR29, so that at least 34 plants per genotype survived until the end of the experiment. None of the traits quantified in this work significantly differed between patches (Wald *χ*^2^ ≤ 1.90, *p* ≥ 0.593) such that “patch” was not considered as a factor in the analyses.

### 2.3. Virus Detection and Quantification

Cucumber mosaic virus was detected and accumulation in leaves was quantified by RT-qPCR in each individual plant. Total RNA was extracted from rosette disk samples using NZYol^®^ (nzytech, Lisbon, Portugal), and 10 ng of total RNA was added to the Brilliant III Ultra-Fast SYBR Green RT-qPCR Master Mix (Agilent Technologies, Santa Clara, CA, USA) according to the manufacturer’s recommendations. Specific primers were used to amplify a 106 nt fragment of the CMV coat protein gene [59]. Each plant sample was assayed in duplicate on a LightCycler 480 II real-time PCR system (Roche). Absolute viral RNA accumulation was quantified as ng of viral RNA per mg of total RNA by comparing with a 10-fold dilution series ranging from 2 × 10^3^ to 2 × 10^7^ ng of purified virion RNA diluted in water, as internal standard.

RT-qPCR CMV detection showed that the success rate of inoculation ranged from 33% for MAR23 to 83% for MAR12 (Table 1). That is, at least eight Lro-CMV-infected replicates per genotype were included in analyses. In addition, between 12% and 50% (for MAR2 and MAR12, respectively) of the mock-inoculated plants were CMV-infected in the field during the experiment (Table 1). No significant correlation between Lro-CMV and field CMV infectivity per plant genotype was observed (*r* = 0.30; *p* = 0.390) (Table 1), suggesting that field-infected CMV prevalence did not depend on the genotype-specific susceptibility. Consequently, analyses were conducted considering all CMV-infected plants, or Lro-CMV and field-infected plants separately.

### 2.4. Effect of CMV Infection on Plant Growth and Reproduction

The effect of virus infection on maximum rosette diameter (*RD*), silique number per plant (*SN*) and total silique length (Fecundity, *Fc*) was quantified by calculating ratios of infected to non-infected plants for each of them, dividing the value of each infected plant by the mean value for the non-infected plants of the same genotype (*Trait_i_/Trait_m_*, where *i* and *m* denote infected and non-infected plants, respectively). Virulence (*V*) was estimated as 1 minus the fecundity ratio, as *Fc* was taken as a proxy to plant fitness.

### 2.5. Plant Tolerance to CMV Infection

Tolerance was quantified as the slope of plant fecundity (*Fc*) to virus accumulation regression, considering both non-infected and infected plants: the shallower the slope, the higher the tolerance. This measure of tolerance can be applied to genotypes rather than to individual plants, which might reduce the power of our approach. Following [70], we confirmed the results of the comparison between slopes using an individual-level variable that also quantifies tolerance: because the slope of a regression measures the change in *Fc* per unit of virus accumulation, we created a new variable (for each plant) dividing *V* by the level of virus multiplication. This variable represents the effect of infection per unit of CMV accumulation, which can be considered as equivalent to the slope of the Arabidopsis fecundity to CMV accumulation regression.

### 2.6. Statistical Analyses

We first analysed the presence of outliers in the distribution of each variable using Grubbs’ test. After removing outliers, *RD* and *RD* ratio was normally distributed, and variances were homogeneous according to Kolmogorov–Smirnov and Levene’s tests, respectively. Therefore, differences between treatments were analysed by general linear models (GLMs). Virus accumulation, individual-level tolerance, *SN*, *Fc* and their different transformations were not normally distributed, and generalised linear models (GzLMs) were used, fitting data to an exponential (*SN*, *Fc*) or log-normal (virus accumulation) distribution according to Akaike’s information criterion (AIC) (R package: rriskDistributions, [71]). Significance of mean differences among classes within each factor were determined by pairwise comparisons using post hoc least significant difference (LSD) analyses (R package: agricolae, [72]). Linear and non-linear models were considered in each bivariate analysis, and the model with the highest *r*-value (correlation coefficient) was chosen as the best at explaining the relationship between each pair of variables. When analysing the relationship between virulence and tolerance, only genotype-level tolerance was used as individual-level tolerance and virulence had high levels of autocorrelation. Statistical analyses were conducted using R v.4.3.0 [73]. For analysing genotype-level tolerance, linear regression equations were compared via ANOVA to test equality of slopes using the statistical software STATGRAPHICS CENTURION 19 (Statgraphics Technologies, The Plains, VA, USA).

## 3. Results

### 3.1. Effect of CMV Infection on Plant Growth and Reproduction Under Natural Conditions

**Figure 1:**
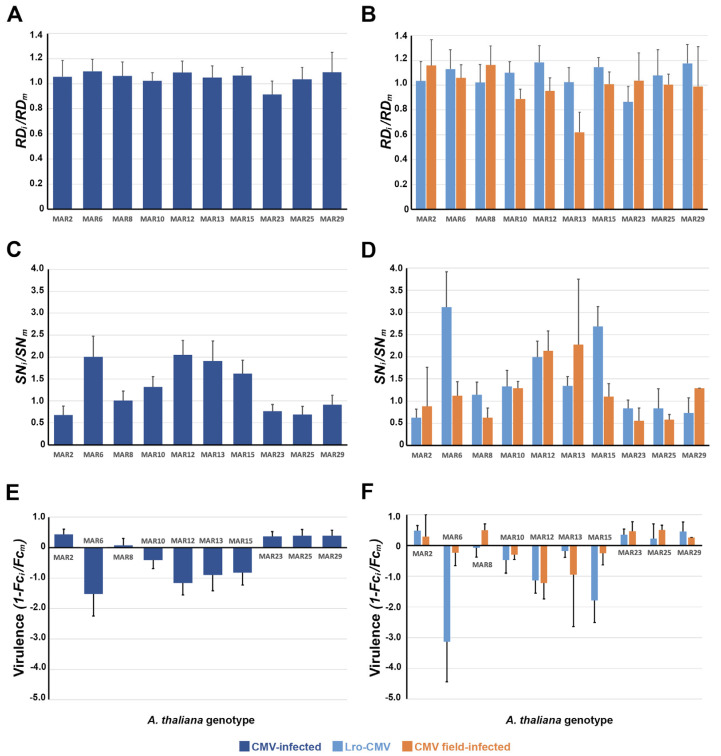
Effect of CMV infection on Arabidopsis growth and reproduction in 10 genotypes grown at the Marjaliza wild Arabidopsis population site. (**A**). Effect of virus infection on maximum rosette diameter (*RD*) per plant genotype pooling together all CMV-infected plants. (**B**). Effect of virus infection on silique number (*SN*) per plant genotype for Lro-infected and field-infected plants, separately. (**C**). Effect of virus infection on *SN* per plant genotype pooling together all CMV-infected plants. (**D**). Effect of virus infection on *SN* per plant genotype for Lro-CMV-infected and field-infected plants, separately. (**E**). CMV virulence per plant genotype pooling together all CMV-infected plants. (**F**). CMV virulence per plant genotype for Lro-CMV-infected and field-infected plants, separately. Data are averages ± standard errors based on 6 to 27 replicates for pooled plants, 6 to 19 replicates for Lro-CMV-infected plants and on 2 to 12 replicates for field-infected plants. Note the different scale for each variable.

We first explored if CMV infection affected plant vegetative growth (as rosette diameter, *RD*) and reproduction (as silique number, *SN*, and total silique length, *Fc*) under natural conditions. For that, we use the infected-to-non-infected ratios of each trait (*RD_i_*/*RD_m_ and SN_i_*/*SN_m_*) or its reciprocal (1–*Fc_i_*/*Fc_m_* as a measure of virulence) (Figure 1).

*RD* ratio did not vary across plant genotypes (*F*_9,150_ = 0.24, *p* = 0.990), with an averaged value across plant genotypes of 1.048 ± 0.160, which indicated that there was no effect of infection on vegetative growth (Figure 1A). In contrast, both *SN* ratio (Wald *χ*^2^_9,139_ = 36.33, *p* = 2 × 10^−6^) and virulence (Wald *χ*^2^_9,139_ = 28.02, *p* = 4.7 × 10^−4^) significantly depended on the plant genotype. MAR6, MAR12, MAR13 and MAR15 showed significantly higher *SN* ratios than the other genotypes (*p* ≤ 0.039), whereas the opposite was observed for MAR2 and MAR25 (*p* ≤ 0.044) (Figure 1C). This trait varied from 0.68 ± 0.20 in MAR2 to 2.05 ± 0.33 in MAR12, indicating that CMV infection can be either detrimental for *SN* or promote silique production depending on the Arabidopsis genotype. Mirroring *SN* ratio, CMV virulence was lower in MAR6, MAR10, MAR12, MAR13 and MAR15 than in the other genotypes (*p* ≤ 0.049) (Figure 1E). Virulence ranged from −1.56 in MAR6 (higher fecundity in infected than in non-infected plants) to 0.44 in MAR2 (negative effect of CMV on *Fc*), again indicating that the effect of CMV infection on Arabidopsis fecundity moves along a parasitism–mutualism continuum. We were aware that a fraction of the CMV-infected plants was not infected with Lro-CMV, but field infected with one or various uncharacterised CMV isolates. Thus, we compared the effect of infection on *RD*, *SN* and *Fc* between Lro-CMV-infected and field-infected plants (Figure 1B,D,F). None of these traits showed differences between the two types of plants (*F*_1,150_ = 0.74, *p* = 0.392 for *RD* ratio; Wald *χ*^2^_1,139_ ≤ 0.15, *p* ≥ 0.693, for the other two traits); the interaction of this factor with plant genotype was not significant (*F*_9,150_ = 0.56, *p* = 0.809; Wald *χ*^2^_9,139_ ≤ 13.69, *p* ≥ 0.090). Hence, the effect of infection on plant growth and reproduction was not dependent on the CMV isolate.

As our results showed that CMV infection affected Arabidopsis fecundity in a genotype-specific manner, we next explored if the analysed genotypes also displayed phenotypic variation in the most important plant defence mechanisms: resistance and tolerance.

### 3.2. CMV Multiplication in A. thaliana Under Natural Conditions

**Figure 2:**
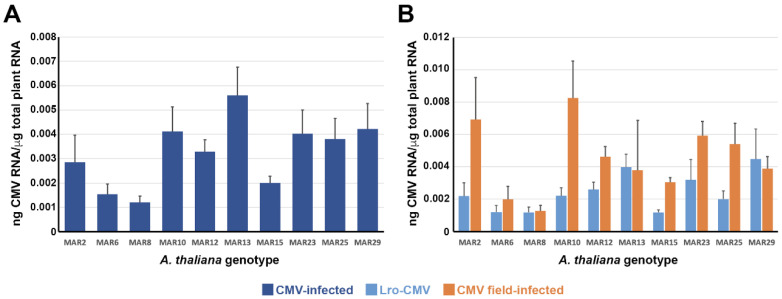
CMV multiplication in 10 Arabidopsis genotypes grown at the Marjaliza wild Arabidopsis population site. (**A**). Virus multiplication per plant genotype pooling together all CMV-infected plants. Data are averages ± standard errors based on 11 to 29 replicates. (**B**). Virus multiplication per plant genotype for Lro-CMV-infected and field-infected plants, separately. Data are averages ± standard errors based on 6 to 19 replicates for Lro-CMV-infected plants and on 2 to 12 replicates for field-infected plants. Note the different scale in each panel.

The level of CMV multiplication was used to evaluate Arabidopsis resistance to virus infection. CMV multiplication was estimated as CMV RNA accumulation in infected leaves (Figure 2A). In general, CMV accumulation was low (average: 0.0033 ± 0.0010 ng of viral RNA/μg of total plant RNA). Generalised linear models indicated that virus accumulation varied according to plant genotype (Wald *χ*^2^_9,140_ = 16.80, *p* = 0.052). The most susceptible genotype was MAR13, which accumulated significantly more viral RNA than MAR2, MAR6, MAR8, MAR12 and MAR15 (*p* ≤ 0.036) (Figure 2A). The most resistant genotype was MAR8, with significantly less viral accumulation than MAR10, MAR12, MAR13, MAR23, MAR25 and MAR29 (*p* ≤ 0.038) (Figure 2A).

We also compared CMV accumulation in Arabidopsis plants infected by Lro-CMV inoculation or in the field (Figure 2B). Interestingly, we observed overall significant differences in virus accumulation (Wald *χ*^2^_1,140_ = 8.18, *p* = 0.004), which was lower for Lro-CMV-infected plants than for field-infected ones (0.0024 ± 0.0004 vs. 0.0045 ± 0.0007) (Figure 2B). However, the virus per plant genotype interaction was not significant (Wald *χ*^2^_1,140_ = 7.01, *p* = 0.636), indicating that Arabidopsis genotypes ranked in the same order for both types of CMV-infected plants. Indeed, a strong linear correlation of CMV multiplication level in the two groups of plants was detected (*r* = 0.71; *p* = 0.050).

Hence, we detected variation in resistance to CMV across plant genotypes under natural conditions. Despite Lro-CMV- and field-infected plants differing in overall virus accumulation, variation in resistance was independent of the virus isolate.

### 3.3. Tolerance to CMV Infection Under Natural Conditions

We analysed if tolerance also varied across plant genotypes. We measure tolerance at the genotype and at the individual plant levels (see Material and Methods, Section 2).

**Figure 3:**
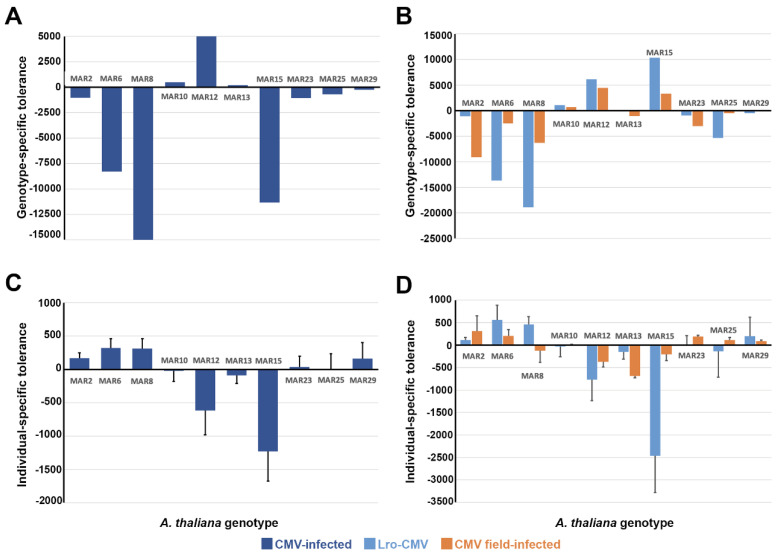
Arabidopsis tolerance to CMV in 10 genotypes grown at the Marjaliza wild population site. (**A**). Genotype-level tolerance (slope of the plant fitness to virus multiplication regression) per plant genotype pooling together all CMV-infected plants. (**B**). Genotype-level tolerance per plant genotype for Lro-CMV-infected and field-infected plants, separately. (**C**). Individual-level tolerance (ratio of virulence to virus multiplication) per plant genotype pooling together all CMV-infected plants. (**D**). Individual-level tolerance per plant genotype for Lro-CMV-infected and field-infected plants, separately. Data are averages ± standard errors based on 6 to 19 replicates for Lro-CMV-infected plants and on 2 to 12 replicates for field-infected plants. Note the different scale in each panel.

When measured at the genotype level, tolerance to CMV varied according to the plant genotype (*F*_9,140_ = 3.40, *p* = 9 × 10^−4^), with MAR6, MAR8 and MAR15 having the steepest negative slope of *Fc* to virus accumulation regression (i.e., being less tolerant to infection). On the other extreme, MAR12 had a positive slope, indicating that infection increases *Fc* as compared to non-infected plants, i.e., overcompensation (Figure 3A). Similar significant differences were obtained with Lro-CMV-infected (*F*_9,89_ = 2.87, *p* = 5.7 × 10^−3^) and field-infected (*F*_9,51_ = 2.02, *p* = 0.051) plants when analysed separately (Figure 3B). No differences in tolerance at the genotype level were detected between the two types of infected plants for any of the plant genotypes (*F* ≤ 1.36, *p* ≥ 0.273). The exception was MAR15, which showed positive *Fc* to virus accumulation slopes in both groups of infected plants, in contrasts with negative values when all plants were pooled together (Figure 3B). Tolerance to CMV at the individual level (ratio between virulence and CMV accumulation) also differed between Arabidopsis genotypes (Wald *χ*^2^_9,101_ = 19.80, *p* = 0.019). MAR6 and MAR8 showed the highest values, indicating that they were the least tolerant to infection, whereas MAR12 and MAR15 showed the lowest values (*p* = 0.025) (Figure 3C). For MAR15, this was unexpected given the negative slope of the genotype-level tolerance. As for the other analysed traits, tolerance at the individual level did not differ according to the type of CMV infection (Lro-CMV inoculations or field infection; Wald *χ*^2^_1,101_ = 0.03, *p* = 0.959); the interaction between plant genotype and type of infection was not significant (Wald *χ*^2^ = 8.82, *p* = 0.454) (Figure 3D). Interestingly, here, both groups of MAR15-infected plants had positive slopes of the *Fc* to virus accumulation relationship and negative values of the virulence-to-virus accumulation ratio as expected. This suggests that the contradictory result of the two tolerance measures observed in MAR15 when all infected plants were analysed together is due to a pooling effect.

Hence, regardless of how Arabidopsis tolerance to CMV was measured, we observed variation for this trait among genotypes under natural conditions, but no effect of the virus isolate.

### 3.4. Relationship Between Plant Defences and Virulence Under Field Conditions

**Figure 4:**
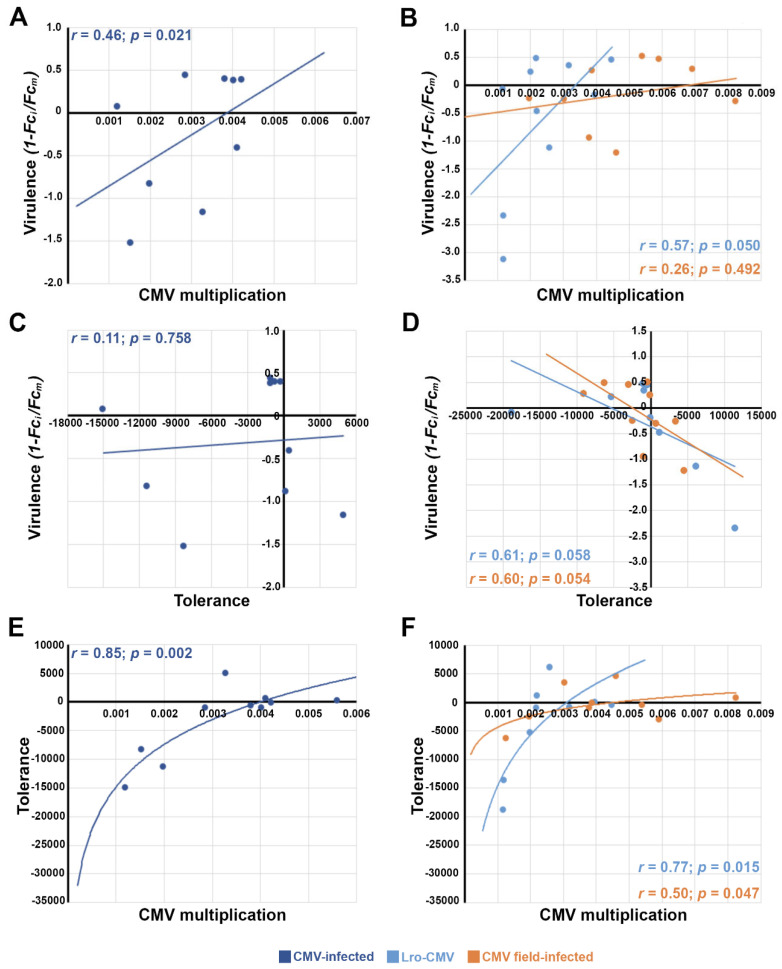
Bivariate relationships between virulence, resistance and tolerance in 10 Arabidopsis genotypes grown at the Marjaliza wild population site. (**A**). Association between CMV accumulation (inverse of resistance) and virulence (effect of infection on *Fc*) pooling together all CMV-infected plants. (**B**). Association between CMV accumulation and virulence for Lro-CMV-infected and field-infected plants, separately. (**C**). Association between genotype-level tolerance (slope of the plant fitness to virus accumulation regression) pooling together all CMV-infected plants. (**D**). Association between genotype-level tolerance and virulence for Lro-CMV-infected and field-infected plants separately. (**E**). Association between genotype-level tolerance and virus accumulation pooling together all CMV-infected plants. (**F**). Association between genotype-level tolerance and virus accumulation for Lro-infected and field-infected plants, separately. Each dot represents values for each plant genotype. Note the different scale in each panel.

The results described above indicate that virulence, and both resistance and tolerance to CMV infection, varied across Arabidopsis genotypes under field conditions. Thus, we studied which of the two plant defences explained better the observed variation in virulence. To do so, we analysed linear and non-linear bivariate relationships between virus accumulation, tolerance and virulence (Figure 4).

We found a positive linear relationship between CMV multiplication and virulence (*r* = 0.46; *p* = 0.021, Figure 4A), that is, virulence and resistance were negatively associated. Such relationship was significant only for Lro-CMV-infected (*r* = 0.57; *p* = 0.050), but not for field-infected (*r* = 0.26; *p* = 0.492) plants when analysed separately (Figure 4B). No significant association between genotype-level tolerance and virulence was observed when all infected plants were analysed together (*r* = 0.11; *p* = 0.758, Figure 4C). In contrast, a significantly negative linear association between the two traits was found in both Lro-CMV- and field-infected plants (*r* ≥ 0.60; *p* ≤ 0.058, Figure 4D). Therefore, for Lro-CMV-infected plants, both tolerance and resistance explain a similar proportion of the variance in virulence, whereas in field-infected plants tolerance appears to be the main plant defence. We also explored the relationship between resistance and tolerance. We found a strong positive logistic association between virus multiplication and tolerance (*r* = 0.85; *p* = 0.002), indicating a negative relationship between resistance and tolerance (Figure 4E). This association held for both Lro-CMV-infected (*r* = 0.77; *p* = 0.015) and field-infected (*r* = 0.50; *p* = 0.047) plants when analysed separately (Figure 4F). These results indicate a concave trade-off between resistance and tolerance.

## 4. Discussion

Understanding the role of resistance and tolerance in plant–virus interactions requires evaluating the effects of virus infection and the expression of host defence traits under field conditions. This is because in nature, viruses may be conditional mutualists of plants [44,51,52], and because both plant gene function and gene effects depend on the environment [53]. In line, studies of the genetic determinants of plant life history traits reported discrepancies between results under controlled and field conditions [18,74,75,76]. This could be the case also for defence traits, as the expression of virus resistance is known to depend on the environment [34,77,78,79]. However, defences against viruses have been analysed mostly under controlled conditions, except for, to our knowledge, two notable studies based on field assays [80,81], which only focused on resistance. Here, we expanded current knowledge to include both resistance and tolerance, and analysed their expression under infection by CMV, a virus not previously considered in these field studies.

We evaluated the response of ten genotypes of Arabidopsis to CMV infection at the site of Marjaliza (MAR) in central Spain, where these genotypes were initially collected. Results show that the effects of infection on the plant fecundity, taken as a proxy to fitness, and the expression of both resistance and tolerance, are genotype-dependent. These results add host genotype to recently identified factors that determine CMV virulence, virus accumulation and effects on different life history traits in two wild Arabidopsis populations, such as the plant population site, cohort and time of infection [44]. Specifically, in the harsh conditions of the MAR Arabidopsis population site, where there is a water deficit during the whole plant growing season, the effect of CMV infection on host growth and fecundity was shown to depend on the plant life cycle: it did not affect seed production in plants of the winter cohort but increased seed production in plants of the spring cohort, regardless of the time of infection relative to the plant development (at the vegetative or reproductive stage) [44]. In the present study, plants were inoculated at the early vegetative growth stage, and transplanted in the field in early February, mimicking a winter cohort. In these conditions, CMV infection had no effect on the plant vegetative growth, estimated by the rosette diameter. In contrast, it had host genotype-specific consequences for plant fecundity, estimated as total silique length, which encompassed negative (i.e., virulence), neutral and positive effects. Therefore, the inclusion of host genotype as a factor indicates that the outcome of the Arabidopsis–CMV interaction can be more plastic than previously shown.

The assayed genotypes also differed in resistance, estimated by the amount of virus accumulation in leaf tissues, and tolerance, whether it was measured at the genotype or at the individual level. Thus, at the conditions of its native habitat, the MAR Arabidopsis population is polymorphic for these traits, mirroring assays of the same genotype under greenhouse conditions [32]. Polymorphisms for Arabidopsis resistance to turnip mosaic virus have been reported to be expressed in the field, but not in the native habitat of the assayed genotypes [80]. Although another study showed that resistance to disease is expressed by Arabidopsis genotypes in their native habitat, the disease-inducing pathogen(s) was not determined [81]. Thus, a major finding of the present work is to show, for the first time, that polymorphisms for virulence, resistance and tolerance to a virus are expressed in the environmental conditions of the native habitat of the assayed plant genotypes.

MAR genotypes expressed tolerance to CMV infection ranging from partial reductions in virulence to higher fitness of infected than non-infected plants (MAR6, MAR12, MAR13, MAR15, Figure 1)—that is, to overcompensation [82]. This phenomenon has been shown to be genotype-dependent in Arabidopsis under herbivory, and after infection by *Hyaloperonospora parasitica* or by CMV [83,84,85], where it occurred in tolerant genotypes and was explained by resource reallocation from growth to reproduction. This is not the case here, as for MAR12 and MAR15, the only tolerant genotypes among the overcompensating ones, there were no effects of infection on vegetative growth, which suggest other, unexplored, mechanisms leading to overcompensation.

Interestingly, the effects of infection and the defence traits were similarly expressed across plant genotypes when inoculated with Lro-CMV and when infected in the field by horizontal transmission with uncharacterised circulating CMV isolates. At odds with these results, earlier work involving three different CMV strains belonging to subgroups I and II of CMV isolates, had identified an effect of virus strain in virulence, resistance and tolerance [64,65]. We have not characterised the CMV isolates circulating in MAR, but they are most probably genetically closely related, as metagenomic analyses showed very limited genetic diversity among CMV isolates infecting different hosts in the same geographic area [63]. Moreover, four CMV isolates, Cdc1-, Cdc2-, Ras- and Lro-CMV, from three different Arabidopsis populations in central Spain show very low levels of genetic diversity between them (0.003 ± 0.001 substitutions/site, Figure S1), and lower than that among isolates from different hosts in central Spain (0.007 ± 0.001 substitutions/site, [63]). Such high-sequence homology could explain the similar plant responses to Lro-CMV and the field CMV isolate(s). It could also be possible that the source of inoculum for field-infected plants were Lro-CMV-infected ones. However, accumulation of the sympatric circulating CMV isolate(s) was higher than that of the allopatric Lro-CMV, which argues against this possibility and may allow the speculation of a local adaptation of the native virus population and/or of the host if tolerance is the preferred defence [86,87]. Compatible with this latter possibility, resistance and tolerance explained a similar fraction of the variance of Lro-CMV virulence, but tolerance was the chief predictor of virulence of the circulating CMV isolates (Figure 4). Last, the higher accumulation of the circulating isolates could also be explained by a decrease in virus titre with increasing time after infection, although our previous work does not support this hypothesis [44,64].

Our results are also relevant in that they provide evidence for two classical hypotheses related to the evolution of virulence in pathogens and of defences in hosts. Thus, according to the trade-off hypothesis of virulence evolution [88,89], a positive correlation between virulence and parasite multiplication is expected, as we find in this study. Also, because of the limitation of host resources, resistance and tolerance are predicted to be negatively correlated across host genotypes [6], as again, we find here. Interestingly, a positive correlation between CMV multiplication in Arabidopsis and virulence, or a negative correlation between resistance and tolerance to CMV across Arabidopsis genotypes was not found in our earlier studies [32,64,65]. In these earlier studies, the Arabidopsis wild genotypes assayed represented the high genetic diversity of the species at large spatial scales, either in Eurasia [64,65] or in the Iberian Peninsula [32], while here they represent the lower diversity at the smaller spatial scale of the MAR population, where all plant genotypes belong to one of the four genetic groups of Arabidopsis in Spain [54]. The comparison of our present and past results strongly suggests that correlations between virus multiplication and virulence, and between resistance and tolerance, in Arabidopsis and possibly other hosts, will only occur within bounded ranges of genetic diversity among plant genotypes. Consequently, these relationships would be dependent on the spatial scale, as is the case for other eco-evolutionary processes pertaining to host–pathogen interactions [90,91,92]. An additional possibility is that the resistance–tolerance trade-off might be apparent only at low levels of virus accumulation as those observed in the field, whereas high levels of virus load under controlled conditions would increase virulence up to a level that overcomes the benefits of tolerance. This behaviour, which has been linked to “constant tolerance” that reduces virulence by a constant factor across the range of pathogen growth rates [87], would blur the resistance–tolerance relationship. In support of this idea, constant tolerance is expected to result in a concave trade-off between resistance and tolerance as that observed here.

## 5. Conclusions

In summary the results of the field experiment presented here show that polymorphisms for the effects of CMV infection and for resistance and tolerance across Arabidopsis wild genotypes are expressed under the environmental conditions of their native habitat. It is highly relevant that these results are consistent with polymorphisms for these traits detected for the same genotypes in assays under controlled greenhouse conditions [32], which strongly suggest that results from greenhouse assays can be the basis of conclusions on the evolution of this and, possibly, other plant–virus interactions. Our results also support hypotheses on the relationships between pathogen multiplication and virulence and between resistance and tolerance, and suggest that these hypotheses may be valid within bounds of host genetic diversity and, consequently, be dependent on the spatial scale, which will require further analyses.

## Figures and Tables

**Table 1:** Infectivity rates of Lro-CMV-inoculated and CMV field-infected plants.

Status	Genotype	N	Infected	Infectivity (%)
Lro-CMV-inoculated				
	MAR2	22	10	45.45
	MAR6	25	12	48.00
	MAR8	28	16	57.14
	MAR10	28	15	53.57
	MAR12	24	20	83.33
	MAR13	27	21	77.77
	MAR15	25	10	40.00
	MAR23	27	10	37.04
	MAR25	24	8	33.33
	MAR29	23	9	39.13
Mock ^1^				
	MAR2	25	3	12.00
	MAR6	28	9	32.14
	MAR8	26	7	26.92
	MAR10	27	9	33.33
	MAR12	26	13	50.00
	MAR13	26	9	34.62
	MAR15	22	10	45.45
	MAR23	22	5	22.73
	MAR25	28	10	35.71
	MAR29	19	8	42.11

^1^ Mock-inoculated plants tested as positive for CMV were considered as field-infected.

## Data Availability

Data is available as Supplementary Material.

## Supplementary Materials

The following supporting information can be downloaded at: https://www.mdpi.com/article/10.3390/v18050494/s1, Table S1: Primers used for the amplification and sequencing of CMV genomic RNAs; Figure S1: Maximum likelihood (ML) phylogenetic relationship of CMV isolates obtained from Arabidopsis wild populations of the Iberian Peninsula with representative isolates of genetically distinct groups. The tree was constructed using the concatenated sequences of the three genomic RNAs of each isolate. ML relationships were inferred using IQTree2 [93], and the nucleotide substitution model GTR + F + G4 as determined by Bayesian information criterion implemented in ModelFinder [94]. Isolates designated in magenta are from subgroup IB, and isolates designated in blue are from subgroup IA. Isolates Ri8 and PV1429 were collected from tomato and zucchini in Spain. Bootstrap percentage values are shown next to the corresponding node. Scale represents genetic distance as number of nucleotide substitutions per site. The tree is midpoint rooted; File S1: Raw data generated in this work.

## Abbreviations

The following abbreviations are used in this manuscript:

*Fc*	Plant fecundity
*RD*	Rosette diameter
*SN*	Silique number
