# Supplementary material for: Polymorphisms for Defence and Virulence in the Arabidopsis thaliana–Cucumber mosaic virus Interaction Are Expressed in the Host’s Native Habitat
Source: Viruses. 2026 Apr 23;18(5):494. doi: 10.3390/v18050494 (PMC13211345; doi:10.3390/v18050494)
Supplement: Supplementary file 1 [file viruses-18-00494-s001.zip › Pagán et al_Supplementary Material.pdf]

Table S1. Primers used for the amplification and sequencing of CMV genomic RNAs.

Name	Sequence (5′-3′)	Location[1]
CMV1+817	AACTGAAGACGTCACACCGT	RNA 1 (817-834)
CMV1 -1471	CGGACGAGTCAACGCTGCAA	RNA 1 (1453-1471)
CMV1+1311	TGTCCATGCAATCTGGCGAGC	RNA 1 (1311-1331)
CMV1-2097	CGGACCTAAACCGCGTTCAT	RNA 1 (2097-2116)
MAAT13	TGGTCTCCTTTTAGAGACCC	RNA 1 (3338-3357)
CMV2+943	TTGTGACTCGACTCTGCCCA	RNA 2 (943-962)
CMV2-1706	GTTATCGGAGCTGGGCATCC	RNA 2 (1706-1723)
CMV2+1517	GCGCTATTTGAGCGCTTCCA	RNA 2 (1517-1536)
CMV2-2512	TTGTGACCTCGTTCCCGTCGAT	RNA 2 (2512-2533)
MAAT12	GTTTATTTACAAGAGCGTACGC	RNA 1 y 2 (1-21)
MAAT14	TGGTCTCCTTTTGGAGGC	RNA 1 y 2 (3030-3046)
MAAT2	CGCGATGCATTGGTCTCCTTTTGGAGGCCC	RNA 3 (2216-2197)
MAAT5	GTAATCTTACCACTGTGTGTGTGCG	RNA 3 (1-25)
FCPB	GGGACCACGGAATCAGACTGGGAG	RNA 3 (700-723)
FCPE	GGAATTCATGGACAAATCTGAATCA	RNA 3 (1252-1262)
CMV2 1003-	GACTGTCGAAGCCTAACATGA	RNA 2 (1033-1054)
CMV2 388+	TGGTTAAACCCATGCGTGTCG	RNA 2 (382-409)

[1] Position in the Fny-CMV genomic nucleotide sequence (NC_002034, NC_002035, NC_001440).

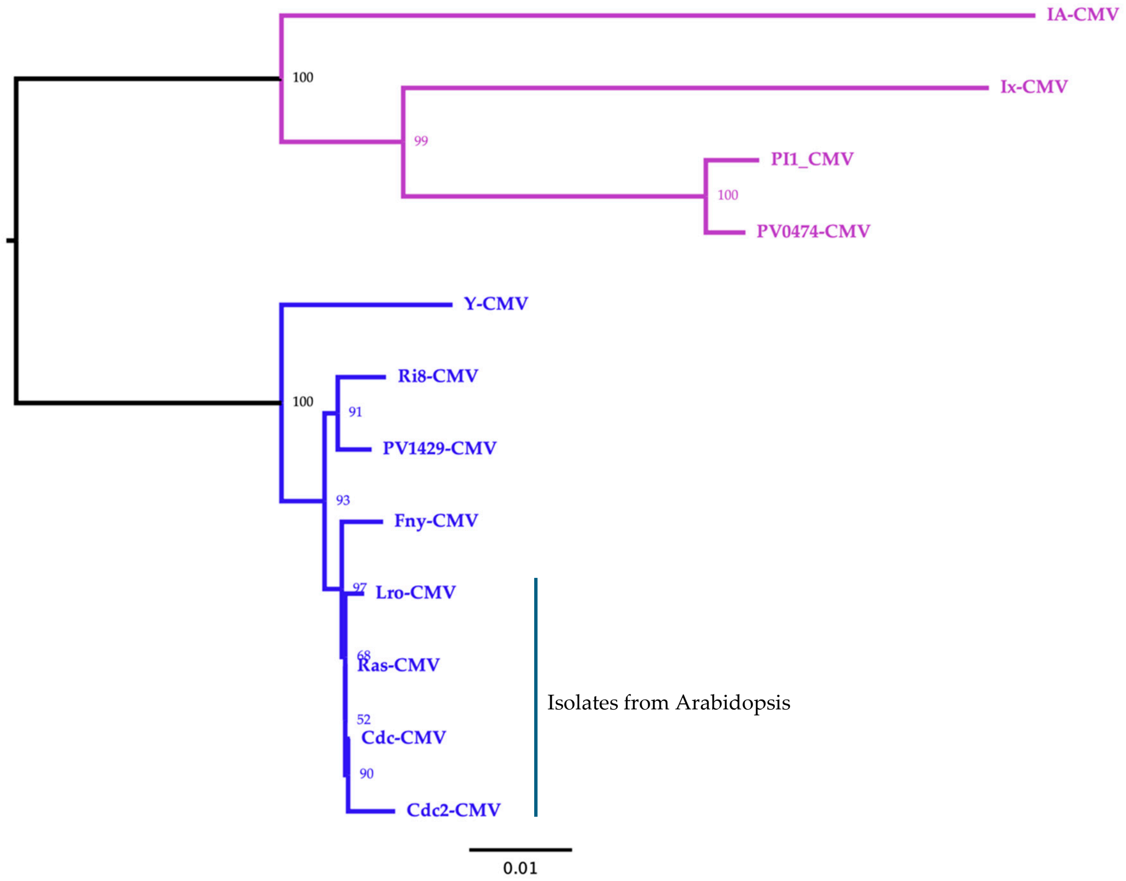

Figure S1. Maximum likelihood (ML) phylogenetic relationship of CMV isolates obtained from Arabidopsis wild populations of the Iberian Peninsula with representative isolates of genetically distinct groups. The tree was constructed using the concatenated sequences of the three genomic RNAs of each isolate. ML relationships were inferred using IQTree2 [93], and the nucleotide substitution model GTR+F+G4 as determined by Bayesian Information Criterion implemented in ModelFinder [94]. Isolates designated in magenta are from subgroup IB, and isolates designated in blue are from subgroup IA. Isolates Ri8 and PV1429 were collected from tomato and zucchini, respectively, in Spain. Bootstrap percentage values are shown next to the corresponding node. Scale represents genetic distance as number of nucleotide substitutions per site. The tree is midpoint rooted.

References

93. Minh, B.Q.; Schmidt, H.A.; Chernomor, O.; Schrempf, D.; Woodhams, M.D.; von Haeseler, A.; Lanfear, R. IQ-TREE 2: New models and efficient methods for phylogenetic inference in the genomic era. *Mol. Biol. Evol.* 2020, 37, 1530-1534.
94. Kalyaanamoorthy, S.; Minh, B.; Wong, T; von Haeseler, A.; Jermiin, L.S. ModelFinder: Fast model selection for accurate phylogenetic estimates. *Nat. Methods* **2017**, *14*, 587-589.
